# P-1369. Impact of the E152K Mutation in Resistance to Taniborbactam and Evolutionary Advantages of NDM-9

**DOI:** 10.1093/ofid/ofae631.1546

**Published:** 2025-01-29

**Authors:** Alejandro J Vila, juliana Delmonti, Francesco G Zuccato, Jazmin Pennesi, Clarisa Parodi, Salvador I Drusin, Diego Moreno, Robert A Bonomo, Lisandro Gonzalez

**Affiliations:** Instituto de Biología Molecular y Celular de Rosario (IBR), Rosario, Santa Fe, Argentina; IBR - University of Rosario, Rosario, Santa Fe, Argentina; IBR - University Of Rosario, Marburg, Hessen, Germany; IBR - University of Rosario, Rosario, Santa Fe, Argentina; Universidad Nacional de Rosario, Rosario, Buenos Aires, Argentina; Instituto de Química Rosrio (IQUIR-CONICET), Rosario, Santa Fe, Argentina; IQUIR, Instituto de Química de Rosario, CONICET, Universidad Nacional de Rosario, Rosario, Santa Fe, Argentina; Case Western Reserve University, Cleveland, Ohio; IBR - University of Rosario, Rosario, Santa Fe, Argentina

## Abstract

**Background:**

NDM is one of the most prevalent carbapenemases worldwide. Boronate-based inhibitors able to target Metallo−β-lactamases, MBLs, such as taniborbactam (TAN) and xeruborbactam (XER), are promising new drugs. However, NDM-9, the third most widespread variant of NDM-1 is refractory to the action of TAN. The E152K substitution in NDM-9 disrupts an attractive electrostatic interaction between the Glu residue in NDM-1 and the side chain of the inhibitor. We aimed to explore how the E152K was selected before the clinical use of TAN and its possible evolutionary pathways.

The C-terminal dynamics that tags protein degradation is quenched in apo-NDM-9

**Figure:**
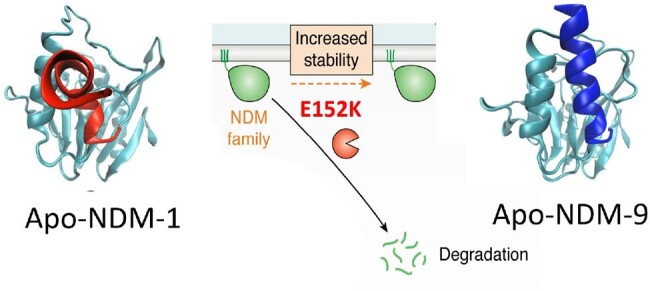

The C-terminal dynamics that tags protein degradation is quenched in apo-NDM-9

**Methods:**

Minimum inhibitory concentrations of ceftazidime in *Escherichia coli* cells expressing NDM variants were determined in Luria Bertani (LB) media with increasing concentrations of dipicolinic acid (DPA). NDM-1 and NDM-9 were expressed in *E. coli* BL21(DE3) pLysS and purified by affinity chromatography. Site-directed mutagenesis was performed. Steady-state catalytic parameters were determined by initial rate measurements and/or by analysis of the progress curves, measured in HEPES buffer, pH 7.5 at 25°C. Deletion mutants in *E. coli* were obtained by CRISPR. NMR spectra were run in a 700 MHz Bruker spectrometer. Docking simulations were performed by using Glide.

**Figure 2.**
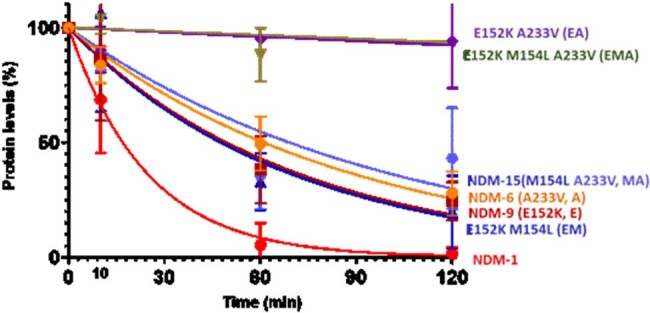
Protein levels in the periplasm of E. coli cells expressing different NDM variants as measured by immunoblotting upon zinc starvation.

**Results:**

NDM-9 is able to confer higher resistance to ceftazidime than NDM-1 under zinc deprivation since apo-NDM-9 is more stable against the periplasmic proteases. NMR reveals that the C-terminal stretch recognized by the protease Prc is more rigid in apo-NDM-9, protecting this variant towards degradation. Despite this, E152K substitution is present only in NDM-9. We obtained new variants of more frequent substitutions such as M154L and A233V. The two double variants and the triple variant were generated. These combinations gave rise to NDM variant hyper-stable against degradation, which also showed resistance to TAN, as supported by docking experiments.

**Conclusion:**

Resistance to TAN exhibited by NDM-9 is a secondary gain-of-function elicited by the E152K substitution, that represents a clinical threat since it stabilizes the protein under zinc limiting conditions. The finding of new combinations on the genetic background of NDM-9 giving resistance to TAN suggests that NDM-9 can further evolve by acquiring more mutations.

**Disclosures:**

**All Authors**: No reported disclosures

